# Low incidence of pelvic sepsis following Hartmann’s procedure for rectal cancer: a retrospective multicentre study

**DOI:** 10.1186/s12893-022-01858-8

**Published:** 2022-12-09

**Authors:** Elin Mariusdottir, Fredrik Jörgren, Amelia Mondlane, Jens Wikström, Marie-Louise Lydrup, Pamela Buchwald

**Affiliations:** 1 Department of Surgery, Helsingborg Hospital, Charlotte Yhlens Gata 10, 25223 Helsingborg, Sweden; 2 Department of Surgery, Skåne University Hospital, Malmö, Sweden; 3 Department of Surgery, Kristianstad Hospital, Kristianstad, Sweden; 4 Lund University, Lund, Sweden

**Keywords:** Rectal cancer, Hartmann’s procedure, Pelvic sepsis, Postoperative complications

## Abstract

**Background:**

Results of previous studies regarding pelvic sepsis after Hartmann’s procedure (HP) for rectal cancer have been inconsistent and few studies report the risk factors. This study aimed to investigate the incidence of pelvic sepsis after HP, identify risk factors and describe when as well as how pelvic sepsis was diagnosed and treated.

**Methods:**

Data were collected from the Swedish Colorectal Cancer Registry on all patients undergoing HP for rectal cancer in the county of Skåne from 2007–2017. Patients diagnosed with pelvic sepsis were compared with patients without pelvic sepsis and risk factors for developing pelvic sepsis were analysed in a multivariable model.

**Results:**

A total of 252 patients were included in the study, with 149 (59%) males, and a median age of 75 years (range 20–92). Altogether, 27 patients (11%) were diagnosed with pelvic sepsis. Risk factors for developing pelvic sepsis were neoadjuvant radiotherapy (OR 7.96, 95% CI 2.54–35.36) and BMI over 25 kg/m^2^ (OR 5.26, 95% CI 1.80–19.50). Median time from operation to diagnosis was 21 days (range 5–355) with 11 (40%) patients diagnosed beyond 30 days postoperatively. The majority of cases 19 (70%) were treated conservatively and none needed major surgery.

**Conclusion:**

Pelvic sepsis occurred in 11% of patients. Neoadjuvant radiotherapy and higher BMI were significant risk factors for developing pelvic sepsis. Forty percent of patients were diagnosed later than 30 days postoperatively and most patients were successfully treated conservatively. Our findings suggest that HP is a valid treatment option for rectal cancer when anastomosis is inappropriate, even in patients receiving neoadjuvant radiotherapy.

## Background

The increasing incidence of rectal cancer, especially among older people, compels surgeons to reconsider the treatment for rectal cancer [1, 2]. The role of HP as a treatment option for rectal cancer has long been debated and its use varies widely between countries [3–5]. According to the Swedish national guidelines, HP is reserved for older, more frail patients as well as those with impaired anal sphincter function [6]. The utilisation of HP has increased in recent years with around 20% of patients with rectal cancer in Sweden undergoing HP [7, 8]. Findings from the Dutch and Norwegian colorectal surgical audits are similar, with an increased use of HP, especially among older patients [9, 10]. However, in the US, the use of HP is rare and, according to the American Society of Colon and Rectal Surgeon’s guidelines, HP is not recommended for rectal cancer [11]. Previous studies have shown a high rate of pelvic sepsis after HP and subsequently advised against HP [12, 13]. Recent studies suggest that the risk of pelvic sepsis may be lower than hitherto assumed [10, 14]. The literature regarding risk factors for pelvic sepsis following HP in rectal cancer is scarce, indicating a need for further studies [15]. A better understanding concerning the prevalence and risk factors of pelvic sepsis is crucial to define the use of HP in rectal cancer treatment.

The primary objective of this study was to investigate the incidence of pelvic sepsis following HP in rectal cancer patients within 1 year postoperatively. Secondary objectives included exploring risk factors for pelvic sepsis as well as the time to diagnosis and the treatment used.

## Methods

### The Swedish Colorectal Cancer Registry

All patients diagnosed with colorectal cancer in Sweden are registered in the Swedish Colorectal Cancer Registry (SCRCR). Data on patient and tumour characteristics, diagnostics, treatment and outcomes are registered in the database. The SCRCR has a coverage of > 99% of patients with rectal cancer and has high validity. A detailed description of the SCRCR has been published [7, 16].

### Inclusion and exclusion criteria

This study is a populations-based, retrospective analysis of patients undergoing HP to treat rectal cancer using prospectively registered data from the SCRCR concerning patients from the county of Skåne from January 1, 2007 to June 30, 2017. The population of Skåne is about 1.3 million persons. Three hospitals perform rectal cancer surgery in the county and data was received from all hospitals. Patients who underwent anterior resection, abdominoperineal resection (APR) or non-abdominal resections were excluded, as were patients with tumour < 5 cm from the anal verge since the Swedish national treatment guidelines recommend abdominoperineal resection in these cases to achieve adequate tumour margins [6]. HP is used in rare cases regarded as exceptions for tumors 0–4 cm from the anal verge, thus excluded from the analysis.

In this study, SCRCR data were expanded with further details from medical charts. Data were obtained regarding preoperative blood tests, smoking, diabetes, and cardiovascular and pulmonary disease. Furthermore, information regarding morbidity associated with the rectal stump, complications within 1 year postoperatively and treatment received was gathered.

### Outcome

Patients that developed pelvic sepsis within 1 year postoperatively were compared with patients without pelvic sepsis in terms of baseline characteristics and postoperative morbidity.

### Definitions

Rectal cancer was defined as adenocarcinoma ≤ 15 cm of the anal verge and low HP was performed if the tumour was located < 10 cm from the anal verge. Emergency surgery was defined as procedure performed within an acute admission often due to bowel perforation, bleeding or bowel obstruction.

Neoadjuvant radiotherapy (RT) was either a short course/immediate surgery, 5 × 5 Gy during the week before surgery, short course/delayed surgery 6–8 weeks after RT or a long course, 2 × 25 Gy with or without chemotherapy followed by surgery 6–8 weeks later.

Pelvic sepsis was defined as abscess formation in the pelvis demonstrated on a CT scan and/or purulent discharge from the anus.

### Statistical analysis

Continuous variables are presented as the median with a range, and categorical data are described using frequencies of counts with associated percentages. Nominal variables were compared between groups using Fisher’s exact test and continuous variables were analysed using a Mann–Whitney test.

To identify risk factors for pelvic sepsis, a univariable logistic regression analysis was carried out. Significant factors from the univariable analysis were included in the multivariable logistic regression to investigate independent risk factors for pelvic sepsis. As the number of patients that developed pelvic sepsis was small it is not advised to include many variables into the multivariable analysis. That could increase the risk of type 1 error as statistically significant differences may not reflect actual differences.

R version 3.6.1 was used for the analysis and a *P* < 0.05 was considered significant.

## Results

### Baseline characteristics and pelvic sepsis

**Fig. 1:**
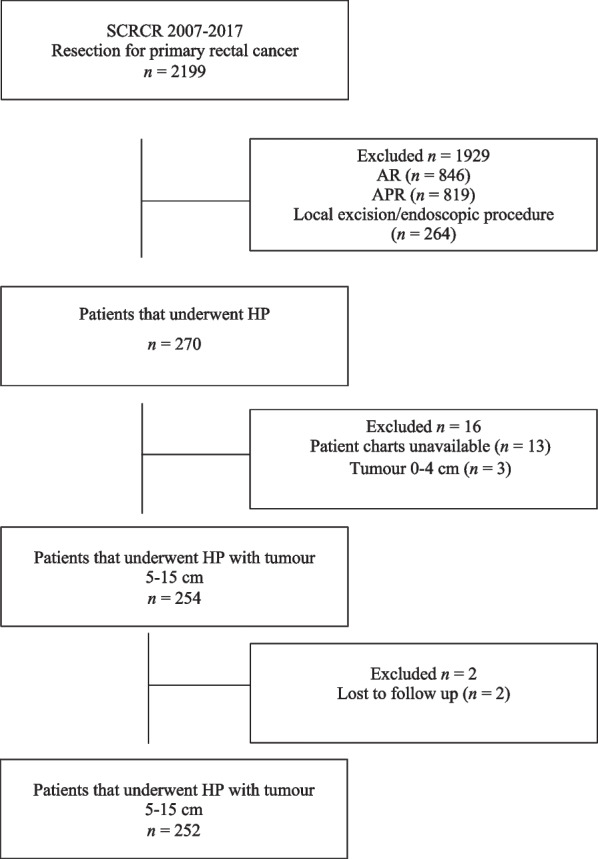
Flow chart of patient selection. Study flow chart of patient selection for Hartmann’s procedure from the Swedish Colorectal Cancer Registry (SCRCR) in the county of Skåne from 2007–2017. *AR* Anterior resection, *APR* Abdominoperineal resection, *HP* Hartmann’s procedure

After exclusions, complete data from 252 consecutive patients who underwent HP for rectal cancer were included in this study (Fig. 1). The majority of patients were operated at Hospital 1 or 177 patients with 66 patients operated at the Hospital 2 and 9 patients at Hospital 3. Demographic data are displayed in Table 1. In 137 (54%) of patients, low HP was performed. Altogether 128 (51%) patients of the study population received neoadjuvant RT and patients treated with RT were more frequently < 75 years, 63% vs. 45% (*P* < 0.0001), respectively.

**Table 1:** Patient characteristics

	All patients (*n* = 252)	Patients with pelvic sepsis (*n* = 27)	Patients without pelvic sepsis (*n* = 225)	*P*-value
Age (years) at surgery*	75 (20–92)	69 (50–85)	76 (20–92)	0.002
Male gender	149 (59)	18 (67)	131 (58)	0.53
BMI > 25 (kg/m^2^)	130 (52)	22 (82)	108 (48)	0.001
Medical history				
Cardiovascular disease	148 (59)	15 (56)	133 (59)	0.68
Diabetes mellitus	40 (16)	7 (26)	33 (15)	0.27
Pulmonary disease	27 (11)	1 (4)	26 (12)	0.32
Immune suppression	13 (5)	0	13 (6)	0.30
Smoking history				0.80
Never	142 (59)	15 (56)	127 (60)	
Former	67 (28)	9 (33)	58 (27)	
Current	31 (13)	3 (11)	28 (13)	
ASA grade 3 or 4	107 (42)	7 (26)	100 (44)	0.07
Albumin (g/L)*	36 (11–48)	37 (23–45)	36 (11–48)	0.47
CEA (µg/L)*	4 (1–465)	10 (1–72)	4 (1–465)	0.35
Creatinine (µmol/L)*	78 (34–235)	81 (47–143)	78 (34–235)	0.38
Tumour height*	10 (5–15)	10 (6–15)	10 (5–15)	0.38
Low 5 cm	6 (2)	0	6 (3)	
Mid 6–10 cm	131 (52)	19 (70)	112 (50)	
High 11–15 cm	115 (46)	8 (30)	107 (47)	
TNM stage				0.52
I	35 (14)	2 (7)	33 (15)	
II	86 (34)	12 (44)	74 (33)	
III	90 (36)	8 (30)	82 (36)	
IV	39 (15)	5 (19)	34 (16)	
Neoadjuvant radiotherapy	128 (51)	22 (81)	106 (47))	< 0.001
Short course/ surgery	51 (20)	12 (44)	39 (17)	
Short course/delayed surgery	25 (10)	1 (4)	24 (11)	
Long course without chemo	6 (2)	2 (7)	4 (1)	
Chemoradiotherapy	46 (20)	7 (26)	39 (19)	0.36
Neoadjuvant chemotherapy	6 (2)	1 (4)	5 (2)	0.47

Patient, tumour and treatment characteristics of patients with rectal cancer treated with Hartmann’s Procedure in the county of Skåne between 2007 and 2017

Values in parenthesis are % unless * where it is median with range

*BMI* body mass index*, CEA* carcinoembryonic antigen. *ASA* American Society of Anaesthesiologists. *TNM* Tumour Node Metastasis staging system

Twenty-seven (11%) patients were diagnosed with pelvic sepsis. Patients diagnosed with pelvic sepsis were younger, 69 years compared to 76 years in patients without pelvic sepsis (*P* = 0.002) and more frequently had BMI over 25 kg/m^2^ (*P* = 0.001). There was no difference in tumour height measured from the anal verge; the median height was 10 cm (Table 1). Altogether, 22 (81%) patients that developed pelvic sepsis had received neoadjuvant RT compared to 106 (47%) of those that did not develop pelvic sepsis (*P* < 0.001).

Table 2 shows the surgical and morbidity data of the study cohort. The operation time was longer 323 min vs. 250 min (*P* = 0.005) and blood loss was higher 750 mL vs. 400 mL in the pelvic sepsis group (*P* = 0.003). The 30-day overall complication rate, including both medical and surgical complications, was higher in the pelvic sepsis group (*P* = 0.006). The reoperation (*P* < 0.003) and readmission (*P* < 0.001) rates were higher in the pelvic sepsis group as well as the length of stay (*P* = 0.04).

**Table 2:** Surgical and outcome data

	All patients (*n* = 252)	Patients with pelvic sepsis (*n* = 27)	Patients without pelvic sepsis (*n* = 225)	*P*-value
Emergency surgery	11 (4)	1 (4)	10 (4)	0.90
Surgical approach				0.80
Open	223 (88)	24 (89)	199 (88)	
Minimally invasive	29 (12)	3 (11)	26 (12)	
Surgical competence				
Colorectal surgeon	206 (82)	22 (81)	184 (82)	0.90
Preoperative bowel preparation	111 (44)	16 (59)	95 (42)	0.15
Operation time (min)*	250 (108–750)	323 (187–750)	246 (108–734)	0.005
Bleeding (mL)*	400 (0–4500)	750 (100–2400)	400 (0–4500)	0.003
Intraoperative perforation	11 (4)	0	11 (5)	0.61
Overall complications^χ^	103 (41)	18 (67)	85 (38)	0.006
Reoperation^χ^	23 (9)	7 (26)	16 (7)	0.003
Re-admission^χ^	22 (9)	8 (30)	14 (6)	< 0.001
Length of stay*	12 (2–143)	17 (5–136)	11 (2–143)	0.04

Surgical and outcome data of patients with rectal cancer treated with Hartmann’s Procedure in the county of Skåne between 2007 and 2017

Values in parenthesis are % unless * where it is median with range. ^χ^Within 30 days

### Risk factors for pelvic sepsis

All clinically relevant variables were tested using univariable logistic regression and findings from the univariable analysis are shown in Table 3.

**Table 3:** Logistic regression analysis

Variables	Univariable analysis			Multivariable analysis		
	OR	95% CI	*P*-value	OR	95% CI	*P*-value
Age (years)	0.95	0.92–0.99	0.008	0.95	0.90–0.99	0.043
Male gender	1.40	0.62–3.42	0.43			
BMI > 25 (kg/m^2^)	5.39	1.95–18.57	0.003	5.26	1.80–19.50	0.001
ASA grade 3 or 4	2.34	0.99–6.17	0.06			
Preoperative radiotherapy	4.94	1.95–15.16	0.002	7.96	2.54–35.36	0.001
Operation time (min)	1.00	1.00–1.01	0.007	1.00	0.99–1.00	0.15
Bleeding (mL)	1.00	0.99–1.00	0.07			

Univariable and multivariable logistic regression analysis of risk factors for the development of pelvic sepsis after HP for rectal cancer

*BMI* body mass index. *ASA* American Society of Anaesthesiologists

In multivariable logistic regression analysis neoadjuvant radiotherapy was identified as a risk factor for pelvic sepsis; OR 7.96 (95% CI 2.54–35.36, *P* = 0.001) as well as BMI over 25 kg/m^2^; OR 5.26 (95% CI 1.80–19.50, *P* = 0.001). Older patients had lower risk of developing pelvic sepsis OR 0.95 (95% CI 0.90–0.99, *P* = 0.043) (Table 3).

### Diagnosis and treatment of pelvis sepsis

The median time from operation to the diagnosis of pelvic sepsis was 21 days (range 5–355 days) with 11 (40%) of patients diagnosed > 30 days postoperatively, seven of these being diagnosed between 30–90 days postoperatively.

Most cases 19 (70%) were diagnosed with CT scan. In the majority of cases, the abscess was located above the stapled rectum and a rectal defect could be palpated with pus draining from the rectum. Nineteen (70%) cases were treated conservatively, with passive rectal drainage, irrigation and antibiotic treatment classified as Clavien–Dindo grade IIIa. Re-operation was needed in 8 (30%) cases with active transrectal drainage via the placement of a tube or a Foley catheter, classified as Clavien–Dindo IIIb. No patient underwent relaparotomy or perineal proctectomy.

### Morbidity and mortality after Hartmann’s procedure

Of the 252 patients included in the study 39 (15%) reported symptoms from the rectal stump, including secretion in 32 (13%) patients, rectal bleeding in 6 (2%) and proctitis in 4 (2%). These symptoms were diagnosed during follow-up and no patient was re-admitted or received in hospital treatment for these complaints.

The 30- and 90-day mortality were 2.8% and 4.7%. None of the patients that developed pelvic sepsis died within 90-days postoperatively.

## Discussion

The current study includes patients undergoing HP for rectal cancer in the county of Skåne with a 1-year follow-up. The incidence of pelvic sepsis was 11%, with 40% of the patients diagnosed more than 30 days after surgery. Neoadjuvant RT and BMI over 25 kg/m^2^ were risk factors for pelvic sepsis in the multivariable analysis, whereas older patients were at a slightly lower risk of developing pelvic sepsis. Most pelvic sepsis patients were treated conservatively and no patient required major surgery. Pelvic sepsis was not more frequent after low HP.

Recent studies have reported a pelvic sepsis rate of 6–8% within 30 days [10, 14, 16]. Our study includes the diagnosis of pelvic sepsis up to 1 year postoperatively with 40% diagnosed later than 30 days postoperatively, and is thus in accordance with these studies. Several small retrospective studies with a longer follow-up time have been inconsistent and report a rate of pelvic sepsis from 10 to 33% [4, 5, 12, 13]. Tottrup et al. [12] concluded a 1 year incidence of pelvic sepsis of 18.6% and 33% if the resection level was low i.e. on the pelvic floor, attributed to poor healing properties of the lower rectum causing dehiscence of the short anorectal stump. However, apart from rectal cancer, the study included patients with diverticulitis as well as other diagnoses which may have influenced the results [12]. Molina et al. compared low HP with APR for distal rectal cancer and found a pelvic sepsis rate of 12.2% after low HP, advocating that APR should be considered in distal rectal cancer when anastomosis is unsuitable [13]. Since APR is associated with impaired wound healing in the perineum [17, 18] intersphincteric APR has been proposed as an alternative to HP. A randomised study, the HAPIrect, has been initiated in Sweden to compare low HP with intersphincteric APR [19].

The present study showed that neoadjuvant RT increased the risk of pelvic sepsis after HP, in line with previous studies [14, 15, 20, 21] indicating that neoadjuvant RT causes defective wound healing and subsequently increases the risk of pelvic sepsis. Although neoadjuvant RT was a risk factor for pelvic sepsis development in a large study from the Dutch surgical audit it did not affect the overall reintervention rate or mortality [15]. Since neoadjuvant RT has been shown to decrease local recurrence, the use of neoadjuvant RT is steadily increasing with around 60% of patients in Sweden receiving RT and over 90% of patients in the Netherlands receiving RT, which may affect postoperative morbidity [22].

Interestingly the ASA grade was lower in the pelvic sepsis group and the pelvic sepsis group was significantly younger. This could be explained by significantly higher RT rate in patients younger than 75 years of age. The increased operation time and blood loss in the pelvic sepsis patients may suggest that technical difficulties occurred intraoperatively. Lastly the rate of overall complications and reoperations were higher in the pelvic sepsis group suggesting that neoadjuvant RT may be a confounding factor, causing difficulties intraoperatively as well as impaired wound healing. This can explain why older patients had a slightly lower risk of developing pelvic sepsis. A contributing factor could be unintended HP in younger patients due to intraoperative problems. As there was no difference in terms of TNM stage, with approximately 50% of patients classified as TNM stage I–II in patients with and without pelvic sepsis, a more advanced tumour stage probably did not affect the results.

Obesity has been shown to increase the risk of postoperative complications such as surgical site infections and respiratory complications [23]. Furthermore, surgeons are less likely to attempt minimally invasive surgery in obese patients and there is a higher risk of conversion in some studies [24]. The present study identified BMI higher than 25 kg/m^2^ as a risk factor for pelvic sepsis. This is in line with a recent study by Jonker et al. [15], which showed that BMI over 30 kg/m^2^ increased the risk for pelvic sepsis.

The fact that 11 patients (4%) underwent emergency surgery for rectal cancer warrants discussion as rectal cancer surgery is seldom urgent. If there is need for emergency surgery HP is often the procedure of choice. Around 0.7% of patients in Sweden undergo emergency surgery, almost exclusively HP. Only 1 patient developed pelvic sepsis after emergency surgery in the present study.

When evaluating the consequences of pelvic sepsis, the present study showed that most patients with pelvic sepsis were successfully treated conservatively, none required major surgery and no patient that developed pelvic sepsis died within 90 days. The 30- and 90-day mortality was consistent with other studies on patients undergoing HP [10, 14]. However, when comparing HP mortality with overall mortality after rectal cancer surgery, the numbers are high, possibly reflecting a selected group of patients undergoing HP [25]. Not many studies report morbidity after HP; the most common complaint in our study was chronic secretion from the anorectal remnant seen in 32 patients (13%). Similar findings are reported by Popiolek et al. [26].

This study was not without limitations; its partially retrospective nature and the relatively small patient cohort should be considered when interpreting the results. Since data on rectal stump length as well as data on partial vs. total mesorectal excision was not recorded in the SCRCR during the study period, low HP was presumed when tumour height was registered < 10 cm and used as a surrogate to predict the level of transection. Furthermore, intersphincteric APR was not registered during the study period, information regarding this alternative to HP could therefore not be gathered. Previous pelvic surgery could affect the risk of developing pelvic sepsis, unfortunately this information was not available in the current study. The strengths of the study were a meticulous review of medical charts and a long follow-up time in a population-based cohort resulting in a complete data set.

There is an ongoing discussion about the best treatment for distal rectal cancer [17, 27]. A better understanding of the incidence of pelvic sepsis and risk factors is desired when constructing treatment recommendations regarding the use of HP in rectal cancer patients. Larger patient cohorts would allow potential risk factors such as smoking or diabetes to be explored, as well as preoperative nutritional status. Other HP aspects such as how many patients undergo unintentional HP as a result of intraoperative adverse events, overall postoperative HP complications and oncological results are issues that warrant addressing.

## Conclusions

In conclusion, this study found the incidence of pelvic sepsis after HP for rectal cancer to be low (11%) after a 1-year follow-up. Surgeons should be aware that almost half of patients are diagnosed beyond 30 days postoperatively. Neoadjuvant RT was the most important risk factor for pelvic sepsis and may be related to difficulties intraoperatively that increase the operation time and the risk of overall complications. Our findings suggest that HP is a valid treatment option for rectal cancer when anastomosis is inappropriate, even in patients receiving neoadjuvant RT.

## Data Availability

The datasets used during the current study are available from the corresponding author on reasonable request.

## Abbreviations

HP: Hartmann’s procedure
SCRCR: Swedish Colorectal Cancer Registry
BMI: Body mass index
OR: Odds ratio
CI: Confidence interval
APR: Abdominoperineal resection
RT: Radiotherapy
TNM: Tumor Node Metastases staging system
